# Detection of serum MCP-1 and TGF-β1 in polymyositis/dermatomyositis patients and its significance

**DOI:** 10.1186/s40001-019-0368-7

**Published:** 2019-02-14

**Authors:** Chun-Ye Wu, Li Li, Li-Hua Zhang

**Affiliations:** Department of Immunology, No. 983 of the Chinese People’s Liberation Army Joint Support Force, No. 60 of Huangwei Street, Hebei District, Tianjin, 300142 China

**Keywords:** PM/DM, MCP-1, TGF-β1, ILD

## Abstract

**Objective:**

This study aims to detect serum levels of monocyte chemoattractant protein-1 (MPC-1) and transforming growth factor-β1 (TGF-β1) in polymyositis/dermatomyositis (PM/DM) patients complicated with interstitial lung disease (ILD), to reveal the significance of the changes in these levels in the pathogenesis of PM/DM complicated with ILD.

**Methods:**

Serum MCP-1 and TGF-β1 levels in PM/DM patients complicated with ILD, patients with pulmonary infections and normal controls (*n *= 30, each) were detected using enzyme-linked immunosorbent assay (ELISA), and the correlation between PM/DM complicated with ILD and serum MCP-1 and TGF-β1 levels was analyzed.

**Results:**

Serum MCP-1 and TGF-β1 levels were both higher in PM/DM patients complicated with ILD compared with patients with pulmonary infections and normal controls.

**Conclusion:**

Serum MCP-1 and TGF-β1 levels increased in PM/DM patients, and were closely correlated to the complication of ILD. This finding can be used for distinguishing between pulmonary infections and ILD, providing a new diagnostic method for the early prediction of DM/PM complicated with ILD.

## Introduction

Polymyositis (PM) and dermatomyositis (DM) are a group of unexplained inflammatory diseases characterized by lesions on the skeletal muscles and skins [1]. These diseases can be found in multiple organ systems, and the respiratory system is particularly impacted [1]. It has been found that palindromic rheumatism is associated with the development of PM/DM, and also other autoimmune rheumatic diseases, such as rheumatoid arthritis, systemic lupus erythematosus, systemic sclerosis, Sjogren’s syndrome, could also be comorbidities of PM/DM patients [2].

The incidence of interstitial lung disease (ILD) in PM/DM patients is 20–86%, and 80% of deaths due to PM/DM are induced by combined ILD [2]. Therefore, the early diagnosis and treatment of ILD is very important [2]. Monocyte chemoattractant protein-1 (MCP-1) is a member of the C subfamily of chemokines, and has monocyte chemotactic activity [3]. In lung tissues of ILD patients, MCP-1 is expressed in epithelial cells, macrophages and vascular endothelial cells; MCP-1 is involved in inflammation and fibrosis response in the process of pulmonary fibrosis by recruiting and activating monocytes and lymphocytes, and is also involved in the extracellular matrix deposition and changes in normal lung structure by promoting the proliferation of desmocytes, the secretion of collagen and the production of fibrosis-promoting cytokines, which all contribute to the occurrence and development of ILD [3]. Transforming growth factor-β1 (TGF-β1) can promote a number of key steps of pulmonary fibrosis, such as the apoptosis of alveolar epithelial cells, the activation and formation of fiber cells and deposition of the extracellular matrix, increasing the collagen secretion of desmocytes, and inducing abnormalities in the micrangium structure and reactivity, which can cause fibrosis of the skin and viscera [4]. TGF-β1 can directly stimulate vascular endothelial cells and induce neovascularization in the body [5]. The appropriate expression of various subtypes of TGF is essential for maintaining normal embryonic development [5]. Studies have revealed that serum MCP-1 and TGF-β1 levels were higher in PM/DM patients than in normal individuals [6, 7]. In the present study, serum MCP-1 and TGF-β1 levels in PM/DM patients were detected by enzyme-linked immunosorbent assay (ELISA), to investigate the possible role of MCP-1 and TGF-β1 in the pathogenesis of PM/DM complicated with ILD, and to provide a new diagnostic method for the early prediction as well as prognosis of DM/PM complicated with ILD.

## Materials and methods

This study was conducted in accordance with the Declaration of Helsinki. This study was conducted with approval from the Ethics Committee of the 254th Hospital of PLA. Written informed consent was obtained from all participants.

### General information (Table 1)

Thirty patients, who were admitted in the 254th Hospital of PLA and definitely diagnosed with PM/DM complicated with ILD from January 2013 to February 2015, were enrolled into this study. Among these patients, 19 patients were male and 11 patients were female. The age of these patients ranged within 40–75 years old, with an average age of 42.20 ± 2.85 years old. All patients met the international diagnostic criteria of PM/DM. The course of disease of these patients ranged within 1–5 years, with an average course of disease of 2.10 ± 0.32 years. And all patients were definitely diagnosed with pulmonary interstitial lesions, and had dry cough, progressive dyspnea, post-activity chest tightness, shortness of breath, fine rales of the base of double lungs; chest computed tomography (CT) revealed clear reticular changes in both lungs. Healthy subjects matched in age and gender who underwent health examinations were enrolled as the healthy control group. All these subjects had no history of immune diseases, no history of infection and vaccination in the past 3 months, and cardiovascular diseases, central nervous system diseases, liver and kidney diseases, diabetes mellitus and other systemic diseases were excluded. Pulmonary infection group: 30 patients with pulmonary infection matched in age and gender were enrolled. Patients with pulmonary infection refer to patients with bacterial infections (tuberculosis and fungal infections were excluded) confirmed by pathogenic findings and consistent clinical manifestations, on the basis of exclusion of pulmonary interstitial lesions by chest resolution computed tomography (HRCT).

**Table 1:** General data

	Cases	Male/female	Average age
The disease group	30	19/11	42.20 ± 2.85
The healthy control group	30	19/11	41.90 ± 1.95
The pulmonary infection group	30	19/11	42.41 ± 2.79
*P*		*P* > 0.05	*P* > 0.05

There is no significant difference among the three groups in age and sex

Criteria for PM/DM complicated with ILD: (1) the diagnosis met the international diagnostic criteria for PM/DM [7]; (2) diffuse ILD induced by other causes such as infections, heart failure and drug reaction were excluded; (3) patients have dry cough, progressive dyspnea, chest tightness and shortness of breath after activities and fine rales at the bases of double lungs; (4) chest CT (including high-resolution CT) revealed reticular changes in bilateral lungs, nodular exudation, frosted glass-like lesions, pulmonary consolidation, and honeycomb lungs; (5) pulmonary function examination revealed restrictive ventilatory dysfunction or diffuse dysfunction (lower than 80% of the expected value); (6) surgical lung biopsy revealed a result that met the ILD. Cases that met the first and second items plus any two of the other four items or the sixth item were diagnosed as DM complicated with ILD [8–10]. Patients with infectious pneumonia that improved after treatment with antibiotics were excluded. In addition, patients with emphysema or tuberculosis induced by occupation, their environment, drugs and cardiac insufficiency, were excluded. (B) The selected age- and gender-matched normal healthy subjects had no history of immunological diseases, no infection and vaccination history within the past 3 months, and were confirmed to have no cardiovascular disease, diseases of the central nervous system, liver and kidney disease, diabetes and other systemic diseases. (C) Pulmonary infection group: thirty age- and gender-matched patients with pulmonary infections were enrolled. Pulmonary infection patients referred to patients who have the corresponding clinical manifestations, which were confirmed to be bacterial infections (except for tuberculosis and fungal infections) by etiological examination; and chest HRCT did not reveal any pulmonary interstitial lesions. The first choice of treatment for PM/DM is corticosteroids, and is usually the oral use of 1.5–2 mg/kg/day of prednisone, which is orally taken every morning daily. Creatase decreases to normal levels at 6–12 weeks after treatment in most patients. The dose began to slowly reduce when muscle force significantly recovered and creatase returned to normal level. When the dose reduced to the maintenance dose (5–10 mg/day), the medication continued for more than 2 years. For recurrent and severe patients, the addition of immune inhibitors should be timely used. The combined treatment of hormones with immune inhibitors could improve curative efficacy, decrease the dosage of hormones, and prevent adverse reactions. Common immune inhibitors include cyclophosphamide, methotrexate, azathioprine and cyclosporine A.

### Materials

Human MCP-1 and TGF-β1 kits were imported and packaged by Watson Biotech Co. Ltd. (Tianjin, China) (48 wells). All serum samples were preserved in a refrigerator at − 20 °C.

### Experimental methods

The operation steps of ELISA for detecting serum MCP-1 and TGF-β1 levels were in strict accordance with the specifications of the kits.

ELISA detection steps: (1) at 20 min before the experiment, the kit was taken out from the refrigerator, balanced to room temperature 25 °C; (2) standard curve was drawn: eight standard wells were set, 100 μL of the diluent of the specimen was added to each well, 100 μL of standards was added to the first well, after mixing, 100 μL was sucked out with a sampler and moved to the second well; and then such twofold dilution was performed repeatedly until the seventh well, finally, 100 μL was sucked out from the seventh well and discarded, making the volume in each well to 100 μL. The eighth well was set as the blank control. (3) The well with sample to be tested: 100 μL of sample to be tested was added to the well, after fully shaken to mix, the culture plate was placed at 37 °C for 20 min, then fully washed 4–6 times with washing liquid, and dried on filter papers. (4) Fifty microlitre of the primary antibody working fluid was added to each well, the culture plate was placed at 37 °C for 60 min, then fully washed 4–6 times with washing liquid, and dried on the filter paper. (5) One hundred microlitre of enzyme-labeled antibody working fluid was added to each well, the culture plate was placed at 37 °C for 60 min, then fully washed 4–6 times with washing liquid, and dried on the filter paper. (6) One hundred microlitre of substrate working fluid was added to each well, the culture plate was placed in the dark at 37 °C for 5–10 min, and then 50 μL of stop solution was added to each well and shaken to mix, the light absorption value was measured at the wavelength of 492-nm.

### Statistical methods

Statistical analysis was conducted using statistical software SPSS 13.0. All data were expressed as mean ± standard deviation (*x* ± SD). Intergroup comparison among multiple groups was conducted using one-way analysis of variance (ANOVA) in a completely randomized design. Pairwise comparison of significant data was conducted using the Bonferroni test. Comparison between two groups of data was conducted using paired *t* test. The correlation between two variables was analyzed using Spearman’s correlation analysis. The level of significance was set at *α* = 0.05.

## Results

Table 2 shows the significant differences in serum MCP-1 and TGF-β1 levels among these groups. Serum MCP-1 and TGF-β1 levels were significantly higher in PM/DM patients than in subjects in the healthy control group and pulmonary infection group. To further explore the roles of MCP-1 and TGF-β1 in the process of pathological changes in PM/DM complicated with ILD, the investigators analyzed the changes in serum MCP-1 and TGF-β1 levels before and after treatment. Table 3 shows the serum MCP-1 and TGF-β1 levels of the patients with PM/DM and ILD before and after treatment, which are clearly lower after treatment than before treatment.

**Table 2:** Serum MCP-1 and TGF-β1 levels among groups ($\bar{x}\, \pm \,s$)

	Cases	MCP-1	*P* value	TGF-β1	*P* value
The disease group	30	1329.24 ± 12.03	< 0.0001 (compared with the healthy control group); < 0.0001 (compared with the pulmonary infection group)	1148.25 ± 31.24	< 0.0001 (compared with the healthy control group); < 0.0001 (compared with the pulmonary infection group)
The healthy control group	30	214.16 ± 10.45	0.071 (compared with the pulmonary infection group)	199.58 ± 21.24	0.064 (compared with the pulmonary infection group)
The pulmonary infection group	30	324.83 ± 18.42		349.85 ± 24.58

**Table 3:** The changes in serum MCP-1 and TGF-β1 levels before and after treatment

	Case	MCP-1	TGF-β1
Before treatment	30	1329.24 ± 12.03	1148.25 ± 31.24
After treatment	30	617.45 ± 11.93	591.64 ± 20.07
*P*		*P* < 0.05	*P* < 0.05

Changes in serum MCP-1 and TGF-β1 levels after treatment are clearly lower than before treatment

## Discussion

Prognosis of patients with PM/DM complicated with ILD is poor. In addition to lesions of ILD, ILD can further increase the risk of pulmonary infection. Furthermore, weak respiratory muscles in PM/DM patients usually cause secretion retention [11]. The treatment of glucocorticoids or immunosuppressive agents can also promote the occurrence of infections [12]. Therefore, many patients are in a vicious circle of infections and uncontrollable diseases before dying, and these patients finally die of respiratory failure [13–15].

In the current study, we revealed that serum MCP-1 and TGF-β1 levels were significantly higher in PM/DM patients complicated with ILD than in subject in the healthy control group and pulmonary infection group, and the differences in MCP-1 and TGF-β1 levels before and after treatment were statistically significant. A study revealed that the mRNA and protein expression of MCP-1 increased in pulmonary epithelial cells in patients with idiopathic pulmonary fibrosis, and the expression of MCP-1 in serum in patients with idiopathic ILD increased [16]. We found that serum MCP-1 levels were significantly higher in PM/DM patients than in subjects in the healthy control group and pulmonary infection group; therefore, serum MCP-1 level was significantly correlated to PM/DM complicated with ILD. A previous study reported that MCP-1 played an important role in the pathogenic process of PM/DM complicated with ILD [4]. For the diagnosis of ILD, sensitivity and specificity of MCP-1 was 60.7% and 68.2%, respectively [4]. Thus, MCP-1 can be a valuable index for the clinical diagnosis of PM/DM complicated with ILD. In addition, the detection of MCP-1 can be used for distinguishing between ILD and pulmonary infections.

A study revealed that high concentrations of TGF-β1 could inhibit the production of IL-2 through lymphocytes, and reduce inflammatory response [6]. The study suggests that TGF-β1 has a negative regulatory effect on immune response. Therefore, an increase in TGF-β1 level in DM patients may have a certain influence in inhibiting immune response and reducing inflammation in tissues and organs.

In patients with DM/PM complicated with ILD, the interval from the emergence of pulmonary symptoms to the visiting of a doctor is short, which is 5.4 months in average [17]. The conditions are usually serious, hypoxia symptoms are obvious, and in-hospital mortality is up to 40%, which can explain why the prognosis of this disease is poor [17, 18]. Therefore, early detection and early diagnosis would improve the survival rate. At present, the clinical diagnosis for DM or PM patients mainly depends on routine examinations of pulmonary functions, chest HRCT and blood gas analysis; and its confirmation depends on lung biopsy [1]. Therefore, diagnosis rate is low, and misdiagnosis and missed diagnosis can easily occur [1]. This study revealed that serum MCP-1 and TGF-β1 levels were significantly higher in PM/DM patients than in healthy controls and pulmonary infection patients, suggesting that MCP-1 and TGF-β1 can be used for the early prediction, as well as the prognosis of DM/PM complicated with ILD.

Due to the limited source of samples and the short clinical observation time, only a small number of patients were enrolled into this study. The investigators will further expand the enrollment in future clinical studies.

## Abbreviations

MPC-1: monocyte chemoattractant protein-1
TGF-β1: transforming growth factor-β1
PM/DM: polymyositis/dermatomyositis
ILD: interstitial lung disease
ELISA: enzyme-linked immunosorbent assay
HRCT: high-resolution computed tomography
SD: standard deviation
ANOVA: analysis of variance
